# Baseline predictors for 28-day COVID-19 severity and mortality among hospitalized patients: results from the IMPACC study

**DOI:** 10.3389/fmed.2025.1604388

**Published:** 2025-07-04

**Authors:** Jintong Hou, Benjamin Haslund-Gourley, Joann Diray-Arce, Annmarie Hoch, Nadine Rouphael, Patrice M. Becker, Alison D. Augustine, Al Ozonoff, Leying Guan, Steven H. Kleinstein, Bjoern Peters, Elaine Reed, Matt Altman, Charles R. Langelier, Holden Maecker, Seunghee Kim, Ruth R. Montgomery, Florian Krammer, Michael Wilson, Walter Eckalbar, Steven E. Bosinger, Ofer Levy, Hanno Steen, Lindsey B. Rosen, Lindsey R. Baden, Esther Melamed, Lauren I. R. Ehrlich, Grace A. McComsey, Rafick P. Sekaly, Joanna Schaenman, Albert C. Shaw, David A. Hafler, David B. Corry, Farrah Kheradmand, Mark A. Atkinson, Scott C. Brakenridge, Nelson I. Agudelo Higuita, Jordan P. Metcalf, Catherine L. Hough, William B. Messer, Bali Pulendran, Kari C. Nadeau, Mark M. Davis, Ana Fernandez Sesma, Viviana Simon, Monica Kraft, Chris Bime, Carolyn S. Calfee, David J. Erle, IMPACC Network, Lucy F. Robinson, Charles B. Cairns, Elias K. Haddad, Mary Ann Comunale

**Affiliations:** ^1^Department of Microbiology and Immunology/Department of Medicine/Department of Epidemiology & Biostatistics, Drexel University, Philadelphia, PA, United States; ^2^Clinical and Data Coordinating Center (CDCC) Precision Vaccines Program, Boston Children's Hospital, Boston, MA, United States; ^3^Emory School of Medicine, Atlanta, GA, United States; ^4^National Institute of Allergy and Infectious Diseases, National Institute of Health, Bethesda, MD, United States; ^5^Yale School of Public Health, and Yale School of Medicine, New Haven, CT, United States; ^6^La Jolla Institute for Immunology, La Jolla, CA, United States; ^7^David Geffen School of Medicine at the University of California Los Angeles, Los Angeles, CA, United States; ^8^Department of Medicine, Benaroya Research Institute, University of Washington, Seattle, WA, United States; ^9^School of Medicine, University of California San Francisco, San Francisco, CA, United States; ^10^Stanford University School of Medicine, Palo Alto, CA, United States; ^11^Icahn School of Medicine at Mount Sinai, New York, NY, United States; ^12^Precision Vaccines Program, Boston Children's Hospital, Harvard Medical School, Boston, MA, United States; ^13^Brigham and Women's Hospital, Harvard Medical School, Boston, MA, United States; ^14^Department of Neurology/Department of Molecular Biosciences, University of Texas, Austin, TX, United States; ^15^Case Western Reserve University and University Hospitals of Cleveland, Cleveland, OH, United States; ^16^Baylor College of Medicine and the Center for Translational Research on Inflammatory Diseases, Houston, TX, United States; ^17^Department of Pathology, Immunology and Laboratory Medicine/Department of Surgery, University of Florida, Gainesville, FL, United States; ^18^Oklahoma University Health Sciences Center, Oklahoma City, OK, United States; ^19^Department of Medicine, Oregon Health Sciences University, Portland, OR, United States; ^20^Department of Medicine, University of Arizona, Tucson, AZ, United States

**Keywords:** COVID-19, severity, mortality, machine learning, SpO_2_/FiO_2_, TNFRSF11B, ribitol, FGF23

## Abstract

**Introduction:**

The coronavirus disease 2019 (COVID-19) pandemic threatened public health and placed a significant burden on medical resources. The Immunophenotyping Assessment in a COVID-19 Cohort (IMPACC) study collected clinical, demographic, blood cytometry, serum receptor-binding domain (RBD) antibody titers, metabolomics, targeted proteomics, nasal metagenomics, Olink, nasal viral load, autoantibody, SARS-CoV-2 antibody titers, and nasal and peripheral blood mononuclear cell (PBMC) transcriptomics data from patients hospitalized with COVID-19. The aim of this study is to select baseline biomarkers and build predictive models for 28-day in-hospital COVID-19 severity and mortality with most predictive variables while prioritizing routinely collected variables.

**Methods:**

We analyzed 1102 hospitalized COVID-19 participants. We used the lasso and forward selection to select top predictors for severity and mortality, and built predictive models based on balanced training data. We then validated the models on testing data.

**Results:**

Severity was best predicted by the baseline SpO_2_/FiO_2_ ratio obtained from COVID-19 patients (test AUC: 0.874). Adding patient age, BMI, FGF23, IL-6, and LTA to the disease severity prediction model improves the test AUC by an additional 3%. The clinical mortality prediction model using SpO_2_/FiO_2_ ratio, age, and BMI resulted in a test AUC of 0.83. Adding laboratory results such as TNFRSF11B and plasma ribitol count increased the prediction model by 3.5%. The severity and mortality prediction models developed outperform the Sequential Organ Failure Assessment (SOFA) score among inpatients and perform similarly to the SOFA score among ICU patients.

**Conclusion:**

This study identifies clinical data and laboratory biomarkers of COVID-19 severity and mortality using machine learning models. The study identifies SpO_2_/FiO_2_ ratio to be the most important predictor for both severity and mortality. Several biomarkers were identified to modestly improve the predictions. The results also provide a baseline of SARS-CoV-2 infection during the early stages of the coronavirus emergence and can serve as a baseline for future studies that inform how the genetic evolution of the coronavirus affects the host response to new variants.

## 1 Introduction

The coronavirus disease 2019 (COVID-19) has caused a global pandemic. By the end of November 2024, over seven million COVID-19 deaths globally have been reported by the World Health Organization (WHO) (1). It has caused significant stress to the healthcare infrastructure, especially early during the pandemic (2). COVID-19 vaccines have been widely administered; however, vaccine policies vary across the international community, and unvaccinated populations still exist. In the U.S., the percentage of U.S. persons vaccinated with at least one dose was 82%, and 4% in some other areas in the world (3). New leadership at the Centers for Disease Control has announced the removal of COVID-19 vaccinations from the schedule for children and pregnant women. In addition, updated vaccines for 2025–2026 have been approved only for those 65 years or older or those with a preexisting condition. Vaccine availability in the US is currently in a state of flux. It is well known that protection from vaccination wanes over time (4). Waning immunity coupled with recent restrictions on primary vaccine or booster availability in the under-65 population will create an increase in the number of people with no previous exposure or a weakened immune response to SARS-CoV-2. Thus, understanding the natural immune process in naive populations could inform treatments for future hospitalized patients, particularly those who have not had a natural infection or booster in several years. This study is unique in that it was conducted prior to the availability of a SARS-CoV-2 vaccine.

Researchers worldwide have identified many factors associated with COVID-19 outcomes and developed models to predict these outcomes (5–8). For example, the Sequential Organ Failure Assessment (SOFA) score has been used to predict in-hospital mortality (9). Moreover, elevated interleukin-6 (IL-6), associated with the host immune response, has been found to be associated with COVID-19 severity (10). However, developing accurate, easy-to-use models using sparse, convenient, and immediately obtained predictors is essential and has not been sufficiently explored.

The primary objective of this study is to develop predictive models for COVID-19 severity and mortality with the most predictive variables while prioritizing routinely collected variables. The secondary objective is to compare the predictivity value of the model with routinely collected variables with the SOFA score. First, we develop predictive models based on routinely collected features at baseline and compare them to the SOFA score for predicting COVID-19 severity and mortality. Then, we assess the predictive value of adding features from an extensive set of immunologic, virologic, proteomic, metabolomic, and genomic variables collected from the same patients.

We explore other baseline predictors in combination with baseline respiratory status to predict 28-day outcomes on admission when only baseline data is available. We examined over 123,000 baseline variables, including clinical variables, serology, receptor-binding domain (RBD) antibody titers, metabolomics, targeted proteomics, nasal metagenomics, Olink, nasal viral load, autoantibody, SARS-CoV2 antibody titers, nasal and PBMC transcriptomics, and whole blood frequencies measured by CyTOF (mass cytometry or cytometry by time-of-flight) for predicting 28-day COVID-19 severity and mortality. The number of variables for each category is specified in Supplementary Table 3. We further show that SpO_2_/FiO_2_ is superior to the SOFA score in predicting in-hospital 28-day severity and is similar to the SOFA score in predicting 28-day in-hospital mortality among ICU patients.

## 2 Methods

### 2.1 Ethics, study design, and setting

The study followed the Strengthening the Reporting of Observational Studies in Epidemiology (STROBE) guidelines for reporting observational studies (11). The study design and biological sample processing have been previously published (12). The IMPACC study team created five trajectories based on the longitudinal degree of respiratory illness over the first 28 days after admission (13), defined as follows: trajectory 1 = brief length of stay; trajectory 2 = intermediate length of stay; trajectory 3 = intermediate length of stay with discharge limitations; trajectory 4 = prolonged hospitalization; and trajectory 5 = fatal. Severe cases were defined as those with prolonged hospitalization or fatal outcomes by day 28 (trajectory 4 or 5).

### 2.2 Study participants and data collection

Patients who were 18 years and older admitted to 20 US hospitals (affiliated with 15 academic institutions) were enrolled. Only symptomatic cases with confirmed positive SARS-CoV-2 PCR were followed longitudinally. Biologic samples consisted of blood and mid-turbinate nasal swabs on enrollment, day 4, day 7, day 14, day 21, and day 28 post-hospital admission. Specific data elements were acquired via a review of electronic medical records during the inpatient period and participant interviews during the outpatient period (13).

### 2.3 Statistical analysis

R version 4.2.3 was used to perform all data analyses. Dummy variables of categorical variables, not including missing categories, were created for least absolute shrinkage and selection operator (lasso) selection. A feature was removed from data analysis if the percentage of its missing values was >20% of all the individuals in a single dataset. Continuous variables with missing values, including continuous BMI (kg/m^2^), symptom onset to admission days, continuous laboratory test variables, and variables from proteomics, Olink, and viral load datasets, were imputed with missForest (maxiter = 10, ntree = 100) for the training data before balancing the training data. Features used for imputing missing data include clinical features, trajectory group (five categories) (13), and laboratory features if it is a merged dataset.

To ensure balanced training and prevent prediction bias that favors the majority class, training data were balanced by duplicating the data for each outcome class until the sample size for each outcome class reached the least common multiple of the outcome classes of the original dataset. Feature selection was then performed on the balanced training data, and a logistic regression was trained on the balanced training data using the selected features. The training performance was obtained by testing the model on the imputed training data before the dataset was balanced. The model was then tested on unimputed testing data, where observations with missing values in the selected features were removed if the percentage of missing values was <5% in the test data.

We used the lasso regression, where dummy variables were used instead of categorical variables, to select the combination of the top features in a dataset predicting severity and mortality. Akaike information criterion (AIC) based forward selection (categorical variables were used) was used as a complementary method to select the top clinical predictors predicting severity and mortality from clinical and laboratory features. To select robust top routinely collected predictors for the clinical model, we chose predictors determined by both lasso and forward selection. We then retained the selected variables in the model and used lasso to select up to four additional features from merged clinical and laboratory data, as laboratory features were either continuous or binary.

Two-sided two-sample *t*-tests were used to compare continuous variables between independent groups (non-severe vs. severe and alive vs. deceased). Chi-squared tests were used to compare categorical variables between these groups. The DeLong method was used to calculate the confidence intervals (CIs) of the area under the curve (AUC), and the paired DeLong test was used to compare two receiver operating characteristic (ROC) curves (14). A *p*-value < 0.05 was considered statistically significant for all tests.

## 3 Results

### 3.1 Baseline characteristics

From May 2020 to March 2021, 1,164 participants with COVID-19 were enrolled and followed for up to 28 days while hospitalized. One thousand one hundred two of the 1,164 participants with complete baseline SpO_2_/FiO_2_ ratio data from 20 hospitals associated with 15 academic centers were included in this analysis (12). None of the patients were vaccinated before enrollment because vaccines were not widely deployed during the conduct of the study. SpO_2_/FiO_2_ ratio (S/F) has been identified as a non-invasive clinical predictor for COVID-19 outcomes such as imminent ventilatory needs and mortality (15, 16). We classified patients into severe and non-severe cohorts based on the five trajectories defined by the IMPACC study (13). The severe cases were those in either trajectory 4 or 5, which were associated with a longer hospital stay and required more medical resources. Demographics, baseline clinical characteristics including comorbidities and symptoms, and common laboratory examination results by disease severity are provided in Supplementary Table 1. The median age of the 1,102 participants was 59 years [interquartile range (IQR): 49–69]. Of these patients, 669 (61%) were men. By day 28 of hospitalization, 99 (9%) patients died. SpO_2_/FiO_2_ at lowest saturation was 369.68 ± 87.18 for the non-severe cohort and 200.37 ± 111.93 for the severe cohort.

In Supplementary Table 1, severe COVID-19 cases are significantly associated with older age, male sex, Hispanic or Latinx ethnicity, White ethnicity, bilateral lung infiltrates, prolonged symptom onset to hospitalization days, lower SpO_2_/FiO_2_ hypertension, diabetes, chest pain, shortness of breath, lymphocyte count (<500/microliter), reduced platelets (<100,000/ml), higher creatinine level (≥1.5 mg/dL), and body mass index (BMI). The severe cohort has a much smaller proportion of underweight patients. Training/test datasets were created for the machine learning model by randomly selecting 40% of the 1,102 participants from each of the 15 academic centers as testing data (*n* = 444, 119 severe cases including 42 deaths) and using the remaining (*n* = 658, 186 severe cases including 57 deaths) as the training data. Of the selected features, continuous creatinine and platelets contain missing values. The percentages of missing values of the two variables are <5% in both the training and test datasets.

### 3.2 Clinical features predict 28-day in-hospital severity

We selected features from non-invasive clinical features and common laboratory features. Both the lasso and forward feature selection methods selected SpO_2_/FiO_2_ as the top feature, with SpO_2_/FiO_2_ and age being the top two clinical features combined, predicting severity and mortality. We chose categorical BMI (underweight, normal weight, overweight, class 1–2 obesity, class 3 obesity, unknown) as the third non-invasive predictor, as categorical BMI was selected as the next predictor by the forward selection, and it had a greater training AUC than that of the next predictor selected by lasso.

Using logistic regression, SpO_2_/FiO_2_ alone yielded a training AUC of 0.865 (95% CI: 0.8308–0.8992) and a test AUC of 0.874 (95% CI: 0.8345–0.9131). A probability threshold of 0.62(SpO_2_/FiO_2_ = 249) yields training specificity and sensitivity of 90.3% and 73.1%, respectively; test specificity and sensitivity are 90.8% and 69.7%, respectively.

The coefficients of the logistic regression model predicting severe cases, derived from balanced training data, are expressed as 3.835–0.01343 ^*^ SpO_2_/FiO_2_.

Thus, the probability of a case being severe is as follows:


(1) $$\begin{array}{l} \text{Probability of severe = 1/}(\text{1+ }exp(-\text{3}.\text{835}\\ \text{ + 0}.\text{01343 }*\;SpO_{2}/FiO_{2})) \end{array}$$

Adding age and categorical BMI to the SpO2/FiO2 logistic model minimally improved prediction. The new model increased training AUC by 2% (AUC: 0.885, 95% CI: 0.8561–0.9145, sensitivity=79.6%, specificity=81.8%, probability cut-off=0.5) and test AUC by 1% (AUC: 0.884, 95% CI: 0.8465-0.9222, sensitivity=77.3%, specificity=83.4%). The coefficients of the logistic regression predicting severe cases are expressed as follows:


(2) $$\begin{array}{l} 2.05-0.014\;*\;SpO_{2}/FiO_{2}+0.04\;*\;age-1.48\;*\;I_{Underweight}\\ \;-0.59\;*\;I_{Overweight,or\;Class\;1-2\;Obesity\;\left(30-39.9\right)}\\ \;+0.09\;*\;I_{Class\;3\;Obesity\;\left(40+\right)}-1.4\;*\;I_{Unknown}, \end{array}$$

where I = 1 (yes) or 0 (no) is an indicator variable.

When excluding patients with missing BMI, the coefficients are similar and are expressed as follows:


(3) $$\begin{array}{l} 2.03-0.014\;*\;SpO_{2}/FiO_{2}+0.04\;*\;age-1.5\;*\;I_{Underweight}\\ \;-0.59\;*\;I_{Overweight,\;or\;Class\;1-2\;Obesity\;\left(30-39.9\right)}\\ \;+0.1\;*\;I_{Class\;3\;Obesity\;\left(40+\right)}. \end{array}$$

**Figure 1:**
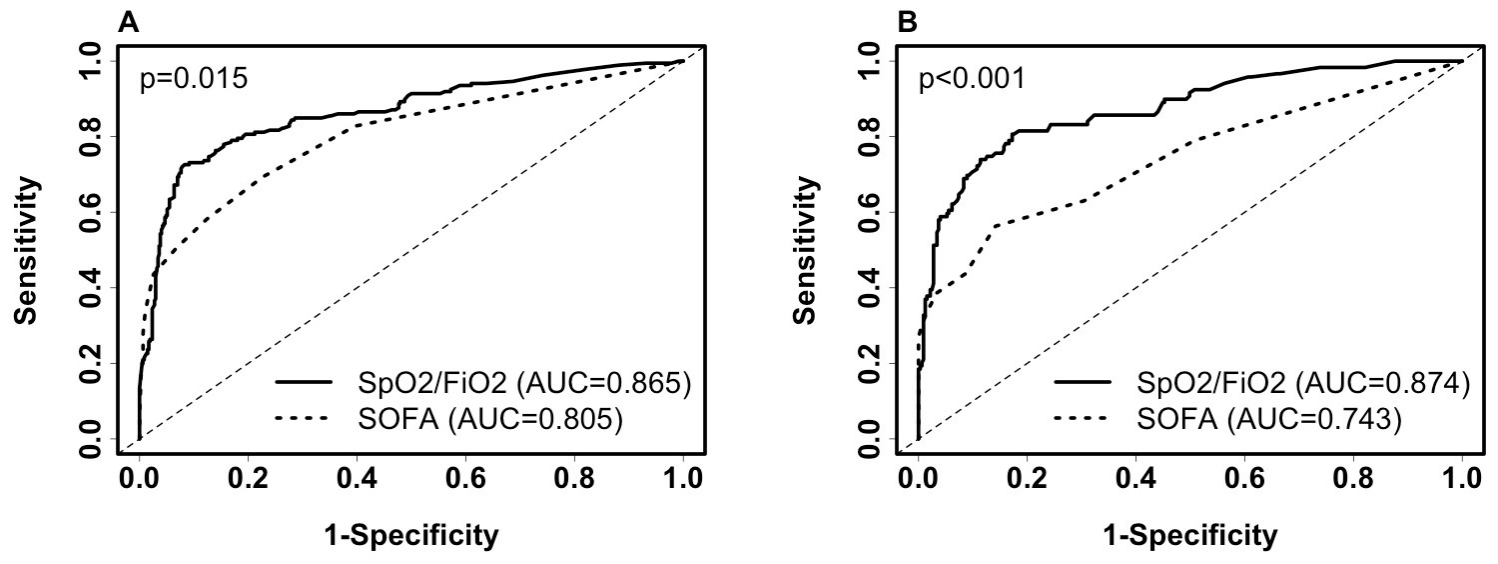
Comparison of receiver operating characteristic (ROC) curves of SpO_2_/FiO_2_ and SOFA for predicting 28-day COVID-19 severity among inpatients. **(A)** ROC on the training set (severe, *n* = 186; non-severe, *n* = 472). SpO_2_/FiO_2_: AUC = 0.865 (95% CI: 0.8308–0.8992, sensitivity = 79.6%, specificity = 81.1%, probability cut-off = 0.5, i.e., SpO_2_/FiO_2_ = 285.5). SOFA score: AUC = 0.805(95% CI: 0.7659–0.8438, sensitivity = 58.1%, specificity = 87.7%). **(B)** ROC on the testing set (severe, *n* = 119; non-severe, *n* = 325). SpO_2_/FiO_2_: AUC = 0.874 (95% CI: 0.8345–0.9131, sensitivity = 78.2%, specificity = 83.4%). SOFA score: AUC = 0.743 (95% CI: 0.6869–0.7985, sensitivity = 56.3%, specificity = 85.8%). Paired Delong's test was used to obtain *p*-values.

To explore the performance of the clinical selector vs. the existing SOFA score approach, we compared SpO_2_/FiO_2_ and the SOFA score in predicting 28-day severity. The SOFA score is calculated based on the number and severity of organ dysfunction in respiratory (PaO_2_/FIO_2_, SaO_2_/FIO_2_), coagulation (platelets), liver (bilirubin), cardiovascular (hypotension), renal (creatinine), and neurologic (coma) organ systems (17). The SOFA score has been used to inform COVID-19 severity and mortality (18, 19). In our dataset, we do not have missing values in the SOFA score. SpO_2_/FiO_2_ outperformed SOFA scores in both training data and testing data (Figure 1. Training AUC: 0.865 vs. 0.805, *p* =0.015. test AUC: 0.874 vs. 0.743, *p* < 0.001). We used SpO_2_/FiO_2_ as the single predictor to compare with SOFA because it is the top non-invasive predictor predicting severity; it alone is significantly more predictive than the SOFA score, while adding age and BMI minimally improves the prediction.

We then compared SpO_2_/FiO_2_ and the SOFA score in predicting 28-day severity among ICU patients at baseline, using the same models derived from ICU and non-ICU patients as described in Equation 1. The AUC for SpO_2_/FiO_2_ was slightly better than that for the SOFA score, but the difference was not statistically significant. Among the 154 ICU patients in the training data, 112 were severe and 42 were non-severe. The AUC for SpO_2_/FiO_2_ was 0.826 (95% CI: 0.7485–0.9029), while the AUC for SOFA score was 0.806 (95% CI: 0.7344–0.8781), with a *p*-value of 0.719. Among the 98 ICU patients in the testing data, 68 were severe and 30 were non-severe. The AUC for SpO_2_/FiO_2_ was 0.778 (95% CI: 0.679–0.8774), and the AUC for the SOFA score was 0.741 (95% CI: 0.6471–0.8358), with a *p*-value of 0.56. When using 0.5 as the probability cut-off, SpO_2_/FiO_2_ showed higher sensitivity and lower specificity than the SOFA score (Supplementary Figure 1).

Next, we examined the sensitivity and specificity discrepancies predicted by SpO_2_/FiO_2_ among ethnicity and sex subgroups, as shown in Supplementary Table 2. When compared with White people, Black people had lower sensitivity (72.7% vs. 79.9%, n_Black_ = 44 vs. n_White_ = 159, *p* = 0.31) and higher specificity (87% vs. 80.8%, n_Black_ = 208 vs. n_White_ = 375, *p* = 0.057), significance was marginal, indicating Black people with severe COVID-19 may appear to have better (higher) SpO_2_/FiO_2_ than White people on admission. Hispanic or Latinx individuals appear to have better sensitivity than non-Hispanic or Latinx (83.8% vs. 74.9%, n_Hispatic_ = 117 vs. n_non − Hispanic_ = 167, *p* = 0.0739). Taken together, there are statistically insignificant differences in sensitivity and specificity among ethnicity, and sex subgroups using SpO_2_/FiO_2_. The insignificant differences may be due to a lack of other less predictive predictors, such as age, ethnicity, sex, other covariates, and randomness of the samples.

### 3.3 The addition of laboratory features improves the predictive capability of the clinical model for severity

**Figure 2:**
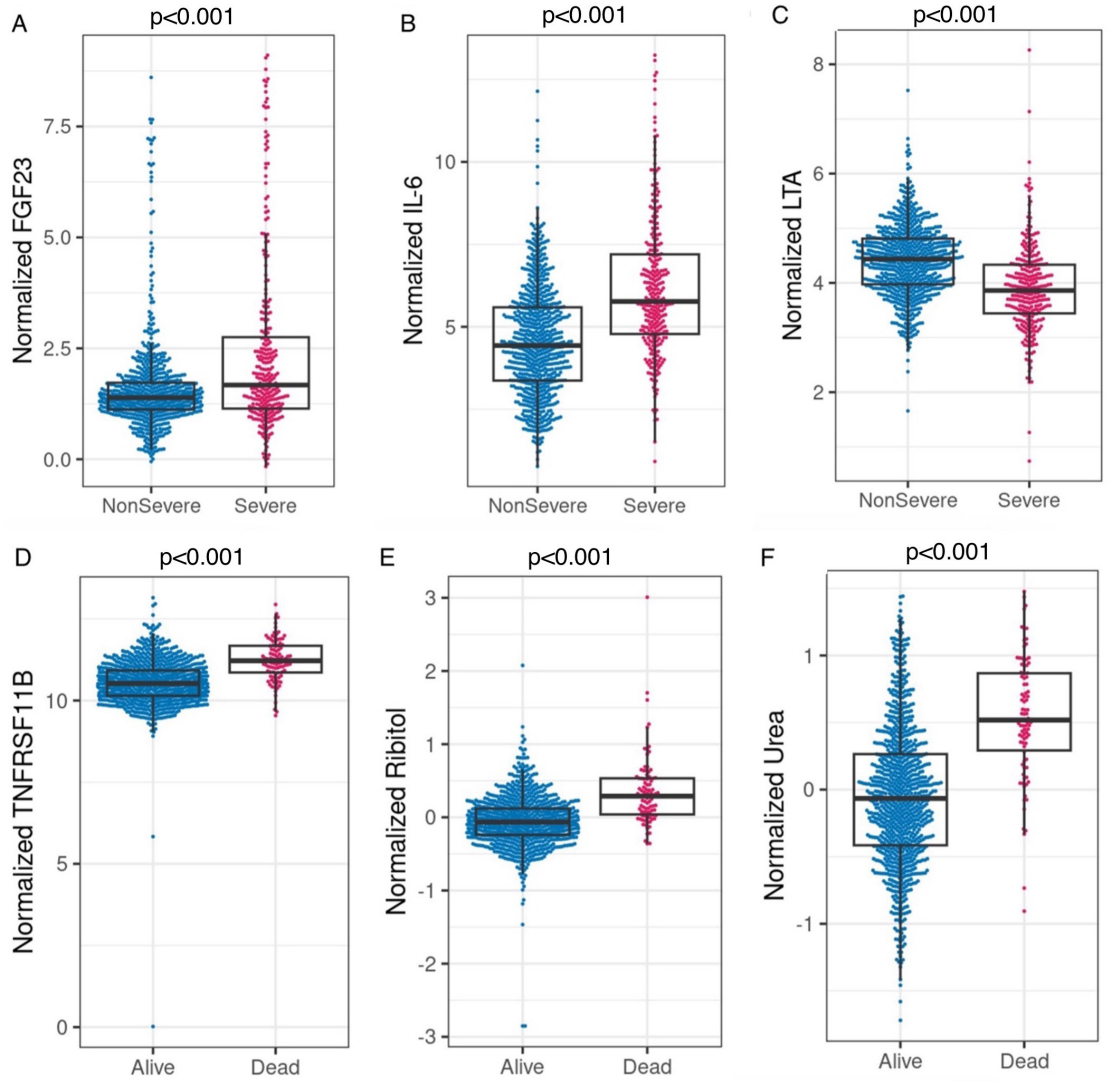
**(A–C)** Comparison of normalized FGF23, IL-6, and LTA between the severe and non-severe cohort in the merged dataset (Olink data merged with clinical data, severe *n* = 292, non-severe *n* = 761). *P* < 0.001 based on two-sample *t*-tests for all three comparisons. **(D–F) Comparison of normalized** TNFRSF11B (Olink feature, alive *n* = 958, Deceased *n* = 95), ribitol, and urea (metabolomics features, alive *n* = 908, deceased *n* = 90) between the deceased and alive cohorts in the merged datasets (merged with the clinical dataset, respectively). *P* < 0.001 based on two-sample *t*-tests for all three comparisons.

**Figure 3:**
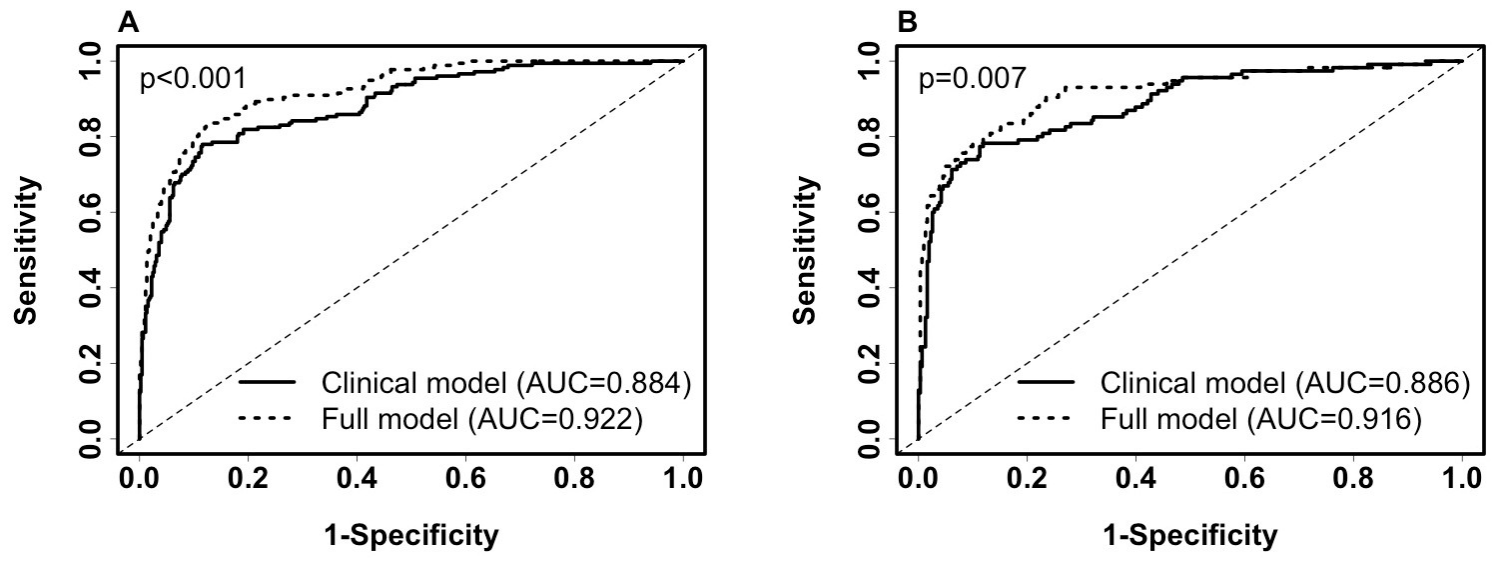
Comparison of ROC curves of the clinical model (SpO_2_/FiO_2_+age+BMI) and the full model (SpO_2_/FiO_2_+age+BMI + FGF23 + IL-6 + LTA) for predicting 28-day in-hospital severity. **(A)**. ROC on the training set (severe, *n* = 177; non-severe, *n* = 450). Clinical model: AUC = 0.884 (95% CI: 0.8539–0.9141, sensitivity = 0.802, specificity = 0.82). Full Model: AUC = 0.922 (95% CI: 0.8993–0.9446, sensitivity = 0.836, specificity = 0.867). **(B)**. ROC on the testing set (severe, *n* = 115; non-severe, *n* = 311). Clinical model: AUC = 0.886 (95% CI: 0.8474–0.9241, sensitivity = 0.783, specificity = 0.83). Full Model: AUC = 0.916 (95% CI: 0.882–0.9491, sensitivity = 0.817, specificity = 0.859). Paired Delong's test was used to obtain *p*-values.

Next, the added predictive power of laboratory features that require a blood draw or sample collection method was assessed. We used the logistic regression model with SpO_2_/FiO_2_, age, and categorical BMI variables as the reference model. In Supplementary Table 3, we merged the clinical dataset, including non-invasive clinical features and common laboratory features, with each of the CyTOF, RBD antibody titers, metabolomics, targeted proteomics, nasal metagenomics, Olink, nasal viral load, autoantibody, SARS-CoV-2 antibody titers, nasal and PBMC transcriptomics datasets representing the expression of 58,302 genes. The laboratory datasets were normalized as detailed in the multi-omics longitudinal study (20). To select sparse features, using the coefficients of the reference model as offset, we used the lasso to select up to four additional predictors from each merged dataset. We built logistic regression models using selected features. We show that adding Fibroblast growth factor 23 (FGF23), IL-6, and Lymphotoxin-alpha (LTA, also known as TNF-β) from the Olink features to the clinical model increased training AUC by 3.8% (*p* < 0.001) and test AUC by 3.1% (*p* = 0.007). Figures 2A–C compares the levels of FGF23, IL-6, and LTA between the severe and non-severe cohorts in the entire merged dataset. Increased FGF23 and IL-6, and decreased LTA levels were associated with severe COVID-19 (*p* < 0.001 for all three comparisons). Figures 3A, B shows ROC curves for full model (SpO_2_/FiO_2_ + age + BMI + FGF23 + IL-6 + LTA, training AUC: 0.922, 95% CI: 0.8993–0.9446; test AUC: 0.916, 95% CI: 0.882–0.9491) are slightly better than that of the clinical model (SpO_2_/FiO_2_ + age + BMI, training AUC: 0.884, 95% CI: 0.8539–0.9141; test AUC: 0.886, 95% CI:0.8474–0.9241), the difference is statistically significant (training *p* < 0.001, test *p* = 0.007).

Adding nasal transcriptomics features or Global Plasma Metabolomics features increased training AUC by at least 4%. However, adding features from remaining datasets did not increase training AUC by more than 3%, and adding laboratory features other than the Olink features did not increase test AUC by more than 1% (Supplementary Table 3).

### 3.4 Clinical features predict 28-day in-hospital mortality

**Figure 4:**
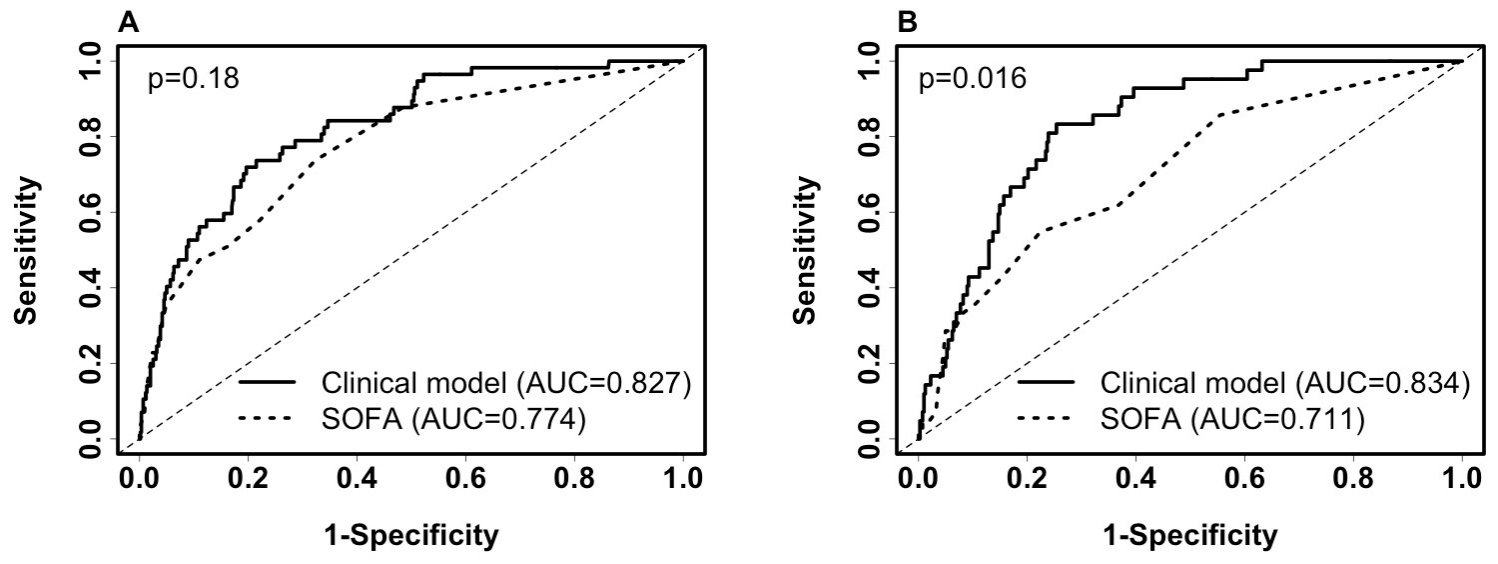
Comparison of ROC curves of the clinical model (SpO_2_/FiO_2_ + age + BMI) and SOFA for predicting 28-day in-hospital mortality. **(A)** ROC on the training set (deceased, *n* = 57; alive, *n* = 601). Clinical model: AUC = 0.827(95% CI: 0.774–0.881, sensitivity = 77.2%, specificity = 73.2%). SOFA score: AUC = 0.774(95% CI: 0.71–0.838, sensitivity = 50.9%, specificity = 83.9%). **(B)** ROC on the testing set (deceased, *n* = 42; Alive, *n* = 402): Clinical model: AUC = 0.834 (95% CI: 0.782–0.887, sensitivity = 81%, specificity = 75.9%). SOFA score: AUC = 0.711 (95% CI: 0.629–0.793, sensitivity = 42.9%, specificity = 84.6%). Paired Delong's test was used to obtain *p*-values.

We identified non-invasive clinical predictors SpO_2_/FiO_2_, age, and BMI combined to be the most predictive of 28-day in-hospital mortality. Table 1 lists the coefficients of the models derived from balanced training data. Confidence intervals of the coefficients are not provided because the training data are balanced. AUCs were obtained from the original training data (*n* = 658, deceased = 57) and testing data (*n* = 444, deceased = 42). SpO_2_/FiO_2_ or age alone had similar predictive capability (AUC 0.7~0.78) and combined to reach an AUC of 0.8. Adding BMI to the model increased AUC by around 2%. Figures 4A, B shows that the clinical model (SpO_2_/FiO_2_, age, and BMI) outperformed the SOFA score predicting 28-day in-hospital mortality, the difference was statistically significant on the testing data (AUC: 0.834 vs. 0.711, *p* = 0.016), and not significant on the training data (AUC: 0.827 vs. 0.774, *p* = 0.18).

**Table 1:** Univariate and multivariable logistic regressions for 28-day in-hospital mortality.

**Model components**	**Model SpO_2_/FiO_2_**	**Model Age**	**Model SpO_2_/FiO_2_ + Age**	**Model SpO_2_/FiO_2_ + Age + BMI**
Training AUC (95%CI, *n* = 658)	0.735 (0.658–0.811)	0.73 (0.67–0.79)	0.802 (0.743–0.862)	0.827 (0.774–0.881)
Test AUC (95%CI, *n* = 444)	0.78 (0.706–0.854)	0.709 (0.634–0.783)	0.82 (0.763–0.878)	0.834 (0.782–0.887)
Intercept	1.834	−4.0018	−2.1237	−1.344
SpO_2_/FiO_2_	−0.00672	-	−0.00636	−0.00688
Age	-	0.0635	0.0608	0.0636
Underweight	-	-	-	−17.46
Overweight	-	-	-	−1.5
Class 1–2 Obesity (30–39.9)	-	-	-	−0.82
Class 3 Obesity (40+)	-	-	-	−0.49
Unknown BMI	-	-	-	−16.8

The reference level of BMI is “normal weight.” Confidence intervals of the coefficient estimates are not provided because the models are derived from the balanced training set.

We then compared the SpO_2_/FiO_2_ + age + BMI model and the SOFA score in predicting 28-day mortality among ICU patients at baseline. The same models derived from ICU and non-ICU patients were used, as shown in Table 1. The AUC of the SpO_2_/FiO_2_ + age + BMI model was slightly better than the AUC of the SOFA score, but the difference was not statistically significant. Among the 154 ICU patients in the training data, 34 were deceased and 120 were alive. The AUC for SpO_2_/FiO_2_ + age + BMI model was 0.766 (95% CI: 0.6753–0.8563), and the AUC for the SOFA score was 0.709 (95% CI: 0.6077–0.8107), with a *p*-value of 0.469. Among the 98 ICU patients in the testing data, 22 were deceased and 76 were alive. The AUC for SpO_2_/FiO_2_ + age + BMI model was 0.69 (95% CI: 0.5737–0.8055), and the AUC for the SOFA score was 0.641 (95% CI: 0.5085–0.7726), with a *p*-value of 0.606. When using 0.5 as the probability cut-off, the SpO_2_/FiO_2_ + age + BMI model had higher sensitivity and lower specificity than the SOFA score (Supplementary Figure 2).

### 3.5 The addition of laboratory features improves the predictive capability of the clinical model for 28-day in-hospital mortality

In Supplementary Table 4, we show that laboratory features improve the predictive capability of the clinical model for mortality in each merged dataset using the SpO_2_/FiO_2_ + age + BMI logistic model as the clinical reference model. We identified that tumor necrosis factor receptor superfamily member 11B (TNFRSF11B) from the Olink features and ribitol from the Global Plasma Metabolomics features increased training AUC by 5.4% and 6.5%, and increased test AUC by 3.5% and 4.6%, respectively. Adding TNFRSF11B to the ribitol + clinical model improved test AUC by < 1%. Adding quinolinate to the ribitol + clinical model increased the training and test AUC by around 1%. Adding creatinine (≥1.5 mg/dL) and platelets to the clinical model increased test AUC by 2.2%, and increased the training and test AUC by 1–2% sequentially. Adding other laboratory features to the clinical model did not increase test AUC by more than 1%. Supplementary Table 5 shows the model parameters for SpO_2_/FiO_2_ + age + BMI + TNFRSF11B, and SpO_2_/FiO_2_ +age+ BMI+ribitol. A higher level of TNFRSF11B or ribitol is associated with higher odds of death after adjusting for SpO_2_/FiO_2_, age, and BMI. The confidence intervals or *p*-values of the model coefficients were not shown because the training data were balanced, so the sample size used for model training was not the same as the original sample size.

### 3.6 Olink and global plasma metabolomics features independently predict 28-day in-hospital mortality

We performed analyses on each merged dataset listed in Supplementary Table 4. Using the lasso and logistic regression, we identified TNFRSF11B as the most predictive feature for mortality among the Olink features and clinical features in the training data. TNFRSF11B alone yielded training AUC 0.803 (95% CI: 0.741–0.865, *n* = 627, deceased *n* = 54, sensitivity: 74.1%, specificity:74%, logistic model parameters: −19.58 + 1.79 ^*^ TNFRSF11B). Its test AUC is 0.762 (95% CI: 0.682–0.842, *n* = 426, deceased *n* = 41, sensitivity: 63.4%, specificity: 76.9%), and is similar to SpO_2_/FiO_2_ (AUC:0.777, 95% CI: 0.702–0.852, *n* = 426, deceased *n* = 41).

We identified urea as the most predictive feature for mortality among the Global Plasma Metabolomics and clinical features in the training data. Urea alone yielded a training AUC 0.823 (95% CI: 0.781–0.873, *n* = 599, deceased *n* = 52, sensitivity: 78.8 %, specificity: 75.1%, logistic model parameters: −0.771 + 2.785 ^*^ urea), and a test AUC 0.778 (95% CI: 0.696–0.86, *n* = 399, deceased *n* = 38, sensitivity: 71.1 %, specificity: 76.5%). Additionally, adding Global Plasma Metabolomics features hydantoin-5-propionate, ribitol, and 3,4-dihydroxybutyrate to the urea only model yielded a training AUC 0.877 (95% CI: 0.844–0.911), and a test AUC 0.837 (95% CI: 0.779–0.896). Figures 2D–F shows that higher levels of TNFRSF11B, plasma ribitol, and plasma urea are associated with higher mortality; *p* < 0.001 for all comparisons.

## 4 Discussion

In this study, we derived easy-to-use prediction formulas for severity and 28-day in-hospital mortality from large-scale clinical, Olink, CyTOF, metabolomics, proteomics, metagenomics, viral load, autoantibody, serum RBD antibody titers, serum SARS-CoV-2 antibody titers, and transcriptomics data collected from 1,102 hospitalized COVID-19 participants prospectively enrolled at 15 study sites with available baseline SpO_2_/FiO_2_ data. Models including easily obtainable clinical features, with and without laboratory biomarkers, were developed. The laboratory biomarkers, which are not immediately available at the bedside to clinicians, provide more prognostic information once results become available. Predictive models were trained on balanced datasets to reduce prediction bias toward the majority class (non-severe, alive). We selected combinations of features that maximize prediction while considering the convenience of obtaining the features. Sparse features selected for predictive models allow for convenient implementation of the tool in practice. While a small number of variables were selected for building models, this does not imply that the deselected variables were not predictive. The deselected variables may still have some predictive power individually or in combination with other features; however, these variables did not improve overall model predictivity given the variables already included. The predictability of deselected variables is not the scope of this study.

SpO_2_/FiO_2_ has been reported to be predictive for respiratory outcomes such as imminent ventilatory use among COVID-19 patients (15). We identified SpO_2_/FiO_2_ as the most predictive predictor for both severity and mortality among routinely collected variables. It is non-invasive, easy, and fast to measure and implement in clinical practice. We explored over 123,000 variables and found that the benefit of adding more predictors is statistically significant but marginal in predicting severity (Figure 3) and mortality, increasing the AUC by 1–2% per added predictor. Therefore, the magnitude of this improvement has limited clinical relevance and may not translate into meaningful clinical benefit or justify the added cost of incorporating these laboratory predictors in a clinical setting. While these predictors may be useful in understanding the natural immunity for COVID-19, it is important to balance statistical significance with clinical utility when translating predictive models for clinical benefit.

Therefore, SpO_2_/FiO_2_ can be used to quickly screen for severe cases in places such as homes or clinics.

The SOFA score has been demonstrated to predict in-hospital mortality among COVID-19 patients and ICU patients (9, 21, 22). However, calculating a SOFA score requires an invasive blood draw; a convenient, non-invasive alternative is desirable. In our data, 28-day in-hospital mortality among severe patients was 1 out of 3. We showed that non-invasive SpO_2_/FiO_2_ was superior to the SOFA score in predicting 28-day severity (*p* < 0.05), thus has the potential to replace the SOFA score in COVID-19-related severity prediction. We showed that the SpO_2_/FiO2 + age + BMI clinical model was better than the SOFA score in predicting 28-day in-hospital mortality among a cohort of hospitalized COVID-19 patients, and similar to the SOFA score in predicting severity and mortality among ICU patients.

We identified that higher IL-6, higher FGF23, and lower lymphotoxin-alpha (LTA) slightly improved the severity prediction by the clinical model. IL-6 stimulates FGF23 production through STAT3 signaling (23). FGF23 regulates cell proliferation and the reabsorption of phosphate by the kidney and is associated with heart failure and impaired host response to infection. Persistent elevated FGF23 in chronic kidney disease has been reported to increase mortality (24–26). Kidney damage in COVID-19 patients is common and ranges in severity. Notably, a bidirectional relationship exists between COVID-19 and kidney disease. Multiple cohort studies identify chronic kidney disease as a risk factor for severe COVID-19, and severe COVID-19 may predispose surviving patients to developing chronic kidney disease (27). A study performed on 85,687 patients reported acute kidney injury due to severe COVID-19 at >20% (28). Our result confirms previous work that circulating FGF23 is associated with acute kidney injury and predicts survival in COVID-19 (29).

In addition, FGF23 has been shown to decrease vitamin D by suppressing 1α-hydroxylase in the kidney (30). Vitamin D can down-regulate pro-inflammatory cytokines, including IL-6 (31). While IL-6 production increases FGF23 and FGF23 functions to reduce vitamin D, it is unclear if low vitamin D levels in COVID-19 are associated with an inability to downregulate IL-6 and prevent a cytokine storm. In addition, while a correlation between vitamin D deficiency and increased COVID-19 severity has been shown, the link has been drawn from indirect association studies (32). Future studies can extend observations to examine the role of vitamin D in the disease course of COVID-19. In addition to IL-6 upregulating the production of FGF23, IL-6 can induce the synthesis of C-reactive protein, serum amyloid A, and other acute-phase proteins. IL-6 also stimulates antibody production and the development of effector T-cells (33). Elevated IL-6 is an endogenous mediator of fever and granulopoiesis and is associated with the induction of a cytokine storm.

LTA, formerly known as tumor necrosis factor-beta (TNF-β), is a pro-inflammatory cytokine and activates the NF-κB pathway. Activation of the NF-κB transcription factor leads to the production of interleukins, including IL-6. It has been suggested that immunomodulation at the level of NF-κB activation and inhibitors of NF-κB degradation may reduce the cytokine storm and thus reduce COVID-19 severity (34). Our results show a decrease in LTA with increased IL-6, suggesting that LTA is not the driver of elevated levels of IL-6 production in COVID-19. It has been reported that viral proteins nsp1, nsp3a, nsp7a, spike, and nucleocapsid protein cause excessive NF-κB activation, possibly contributing to severe disease and high case-fatality rate (34). However, LTA was reported to be involved in eliminating viral infections (35). We show that LTA is significantly lower in the severe group than the non-severe group (*p* < 0.001). Our findings are contrary to a small study that found no significant difference in LTA levels between healthy donors (*n* = 20), COVID-19 survivors (*n* = 13), and COVID-19 non-survivors (*n* = 16) (36).

Our study shows that elevated TNFRSF11B and ribitol improve the prediction of the clinical model for mortality, and urea alone is predictive of mortality. TNFRSF11B level has been reported to be elevated in neuro-COVID cases, which is associated with higher in-hospital mortality (37, 38). Interestingly, increased TNFRSF11B has also been reported to be elevated in the plasma of patients with sepsis–acute respiratory disease syndrome (ARDS) associated with vascular endothelial dysfunction (39). Ribitol is a pentose alcohol formed by the reduction of ribose. Several sugars, including ribitol, are increased in severe COVID-19 cases (40). Our study shows that ribitol improved the prediction of mortality. In addition, ribitol and other pentose-related metabolites have been shown to be higher in male vs. female severe COVID-19 patients (41). In contrast, our study shows that ribitol is lower in men vs. women in severe or deceased COVID-19 patients, but the differences were not significant (Supplementary Table 6). Finally, our results show that urea alone is a strong predictor of mortality. Other studies have associated increased urea level at presentation with COVID-19 predictive of ICU admission and a blood urea nitrogen (BUN; ≥7.37 mmol/L) with increased 28-day mortality and increased admittance to the ICU among COVID-19 patients (42, 43). Therefore, we should consider ribitol, urea, and TNFRSF11B as good predictors of mortality.

This study has several limitations. First, the study sample collection occurred between May 2020 and April 2021, and patients were infected with the original Wuhan or very early variants of SARS-CoV-2 and were unvaccinated. It is unknown if the prediction performance can be generalized to patients infected with more recent variants and patients with hybrid immunity, as the potential differences in immune response and treatment practice may affect outcome model performance. Our study is a multicenter study and does not fully capture the variability that may be encountered in other clinical settings. Future studies are needed to assess its applicability across different variants and external clinical settings. Second, the cohort did not include pregnant women, children, asymptomatic cases, or cases that were not hospitalized (13). Finally, vitamin D level was not measured; thus, the added predictive capability of vitamin D was unclear. However, this study provides a baseline to aid future studies in understanding how the immune system response changes in response to the evolution of new SARS-CoV-2 variants and the impact on treatment practices.

In conclusion, our study suggests that measuring SpO_2_/FiO_2_, age, and BMI can be used as a rapid predictive tool for the severity and mortality associated with COVID-19. In addition, increased levels of IL-6 and FGF23 and decreased levels of LTA improve the prediction of the clinical model for severity. Elevated TNFRSF11B and ribitol improve the prediction of the clinical model for mortality.

## Data Availability

The data analyzed in this study is subject to the following licenses/restrictions: ImmPort (immport.org) under study accession SDY 1760. Requests to access these datasets should be directed to Joann.Arce@childrens.harvard.edu.

## Group members of IMPACC network

**National Institute of Allergy and Infectious Diseases, National Institute of Health, Bethesda, MD**, United States:

Patrice M. Becker, Alison D. Augustine, Steven M. Holland, Lindsey B. Rosen, Serena Lee, Tatyana Vaysman

**Clinical and Data Coordinating Center (CDCC), Precision Vaccines Program, Boston Children's Hospital, Harvard Medical School, Boston, MA**, United States:

Al Ozonoff, Joann Diray-Arce, Jing Chen, Alvin T. Kho, Carly E. Milliren, Annmarie Hoch, Ana C. Chang, Kerry McEnaney, Caitlin Syphurs, Brenda Barton, Claudia Lentucci, Maimouna D. Murphy, Mehmet Saluvan, Tanzia Shaheen, Shanshan Liu, Marisa Albert, Arash Nemati Hayati, Robert Bryant, James Abraham, Mitchell Cooney, Meagan Karoly

**Benaroya Research Institute, University of Washington, Seattle, WA**, United States:

Matthew C. Altman, Naresh Doni Jayavelu, Scott Presnell, Bernard Kohr, Tomasz Jancsyk, Azlann Arnett

**La Jolla Institute for Immunology, La Jolla, CA**, United States:

Bjoern Peters, James A. Overton, Randi Vita, Kerstin Westendorf

**Knocean Inc. Toronto, ON, Canada:**

James A. Overton

**Precision Vaccines Program, Boston Children's Hospital, Harvard Medical School, Boston, MA**, United States:

Ofer Levy, Hanno Steen, Patrick van Zalm, Benoit Fatou, Kinga K. Smolen, Arthur Viode, Simon van Haren, Meenakshi Jha, David Stevenson, Sanya Thomas, Boryana Petrova, Naama Kanarek

**Brigham and Women's Hospital, Harvard Medical School, Boston, MA**, United States:

Lindsey R. Baden, Kevin Mendez, Jessica Lasky-Su, Alexandra Tong, Rebecca Rooks, Michael Desjardins, Amy C. Sherman, Stephen R. Walsh, Xhoi Mitre, Jessica Cauley, Xiaofang Li, Bethany Evans, Christina Montesano, Jose Humberto Licona, Jonathan Krauss, Nicholas C. Issa, Jun Bai Park Chang, Natalie Izaguirre

**Metabolon Inc, Morrisville, NC**, United States:

Scott R. Hutton, Greg Michelotti, Kari Wong

**Prevention of Organ Failure (PROOF) Centre of Excellence, University of British Columbia, Vancouver, BC, Canada:**

Scott J. Tebbutt, Casey P. Shannon

**Case Western Reserve University and University Hospitals of Cleveland, Cleveland, OH**, United States:

Rafick-Pierre Sekaly, Slim Fourati, Grace A. McComsey, Paul Harris, Scott Sieg, George Yendewa, Mary Consolo, Heather Tribout, Susan Pereira Ribeiro

**Drexel University, Tower Health Hospital, Philadelphia, PA**, United States:

Charles B. Cairns, Elias K. Haddad, Michele A. Kutzler, Mariana Bernui, Gina Cusimano, Jennifer Connors, Kyra Woloszczuk, David Joyner, Carolyn Edwards, Edward Lee, Edward Lin, Nataliya Melnyk, Debra L. Powell, James N. Kim, I. Michael Goonewardene, Brent Simmons, Cecilia M. Smith, Mark Martens, Brett Croen, Nicholas C. Semenza, Mathew R. Bell, Sara Furukawa, Renee McLin, George P. Tegos, Brandon Rogowski, Nathan Mege, Kristen Ulring, Pam Schearer, Judie Sheidy, Crystal Nagle

**MyOwnMed Inc., Bethesda, MD**, United States:

Vicki Seyfert-Margolis

**Emory School of Medicine, Atlanta, GA**, United States:

Nadine Rouphael, Steven E. Bosinger, Arun K. Boddapati, Greg K. Tharp, Kathryn L. Pellegrini, Brandi Johnson, Bernadine Panganiban, Christopher Huerta, Evan J. Anderson, Hady Samaha, Jonathan E. Sevransky, Laurel Bristow, Elizabeth Beagle, David Cowan, Sydney Hamilton, Thomas Hodder, Amer Bechnak, Andrew Cheng, Aneesh Mehta, Caroline R. Ciric, Christine Spainhour, Erin Carter, Erin M. Scherer, Jacob Usher, Kieffer Hellmeister, Laila Hussaini, Lauren Hewitt, Nina Mcnair, Susan Pereira Ribeiro, Sonia Wimalasena

**Icahn School of Medicine at Mount Sinai, New York, NY**, United States:

Ana Fernandez-Sesma, Viviana Simon, Florian Krammer, Harm Van Bakel, Seunghee Kim-Schulze, Ana Silvia Gonzalez-Reiche, Jingjing Qi, Brian Lee, Juan Manuel Carreño, Gagandeep Singh, Ariel Raskin, Johnstone Tcheou, Zain Khalil, Adriana van de Guchte, Keith Farrugia, Zenab Khan, Geoffrey Kelly, Komal Srivastava, Lily Q. Eaker, Maria C. Bermúdez-González, Lubbertus C.F. Mulder, Katherine F. Beach, Miti Saksena, Deena Altman, Erna Kojic, Levy A. Sominsky, Arman Azad, Dominika Bielak, Hisaaki Kawabata, Temima Yellin, Miriam Fried, Leeba Sullivan, Sara Morris, Giulio Kleiner, Daniel Stadlbauer, Jayeeta Dutta, Hui Xie, Manishkumar Patel, Kai Nie, Brian Monahan

**Immunai Inc., New York, NY**, United States:

Adeeb Rahman

**Oregon Health & Science University, Portland, OR**, United States:

William B. Messer, Catherine L. Hough, Sarah A.R. Siegel, Peter E. Sullivan, Zhengchun Lu, Amanda E. Brunton, Matthew Strand, Zoe L. Lyski, Felicity J. Coulter, Courtney Micheletti

**Stanford University School of Medicine, Palo Alto, CA**, United States:

Holden Maecker, Bali Pulendran, Kari C. Nadeau, Yael Rosenberg-Hasson, Michael Leipold, Natalia Sigal, Angela Rogers, Andrea Fernandes, Monali Manohar, Evan Do, Iris Chang, Alexandra S. Lee, Catherine Blish, Henna Naz Din, Jonasel Roque, Linda N. Geng, Maja Artandi, Mark M. Davis, Neera Ahuja, Samuel S. Yang, Sharon Chinthrajah, Thomas Hagan, Tyson H. Holmes, Koji Abe

**David Geffen School of Medicine at the University of California Los Angeles, Los Angeles, CA**, United States:

Elaine F. Reed, Joanna Schaenman, Ramin Salehi-Rad, Adreanne M. Rivera, Harry C. Pickering, Subha Sen, David Elashoff, Dawn C. Ward, Jenny Brook, Estefania Ramires-Sanchez, Megan Llamas, Claudia Perdomo, Clara E. Magyar, Jennifer Fulcher

**University of California San Francisco, San Francisco, CA**, United States:

David J. Erle, Carolyn S. Calfee, Carolyn M. Hendrickson, Kirsten N. Kangelaris, Viet Nguyen, Deanna Lee, Suzanna Chak, Rajani Ghale, Ana Gonzalez, Alejandra Jauregui, Carolyn Leroux, Luz Torres Altamirano, Ahmad Sadeed Rashid, Andrew Willmore, Prescott G. Woodruff, Matthew F. Krummel, Sidney Carrillo, Alyssa Ward, Charles R. Langelier, Ravi Patel, Michael Wilson, Ravi Dandekar, Bonny Alvarenga, Jayant Rajan, Walter Eckalbar, Andrew W. Schroeder, Gabriela K. Fragiadakis, Alexandra Tsitsiklis, Eran Mick, Yanedth Sanchez Guerrero, Christina Love, Lenka Maliskova, Michael Adkisson, Aleksandra Leligdowicz, Alexander Beagle, Arjun Rao, Austin Sigman, Bushra Samad, Cindy Curiel, Cole Shaw, Gayelan Tietje-Ulrich, Jeff Milush, Jonathan Singer, Joshua J. Vasquez, Kevin Tang, Legna Betancourt, Lekshmi Santhosh, Logan Pierce, Maria Tecero Paz, Michael Matthay, Neeta Thakur, Nicklaus Rodriguez, Nicole Sutter, Norman Jones, Pratik Sinha, Priya Prasad, Raphael Lota, Saurabh Asthana, Sharvari Bhide, Tasha Lea, Yumiko Abe-Jones

**Yale School of Medicine, New Haven, CT**, United States:

David A. Hafler, Ruth R. Montgomery, Albert C. Shaw, Steven H. Kleinstein, Jeremy P. Gygi, Dylan Duchen, Shrikant Pawar, Anna Konstorum, Ernie Chen, Chris Cotsapas, Xiaomei Wang, Charles Dela Cruz, Akiko Iwasaki, Subhasis Mohanty, Allison Nelson, Yujiao Zhao, Shelli Farhadian, Hiromitsu Asashima, Omkar Chaudhary, Andreas Coppi, John Fournier, M. Catherine Muenker, Khadir Raddassi, Michael Rainone, William Ruff, Syim Salahuddin, Wade L. Shulz, Pavithra Vijayakumar, Haowei Wang, Esio Wunder Jr., H. Patrick Young, Albert I. Ko, Gisela Gabernet

**Yale School of Public Health, New Haven, CT**, United States:

Denise Esserman, Leying Guan, Anderson Brito, Jessica Rothman, Nathan D. Grubaugh, Kexin Wang, Leqi Xu

**Baylor College of Medicine and the Center for Translational Research on Inflammatory Diseases, Houston, TX**, United States:

David B. Corry, Farrah Kheradmand, Li-Zhen Song, Ebony Nelson

**Oklahoma University Health Sciences Center, Oklahoma City, OK**, United States:

Jordan P. Metcalf, Nelson I. Agudelo Higuita, Lauren A. Sinko, J. Leland Booth, Douglas A. Drevets, Brent R. Brown

**University of Arizona, Tucson, AZ**, United States:

Monica Kraft, Chris Bime, Jarrod Mosier, Heidi Erickson, Ron Schunk, Hiroki Kimura, Michelle Conway, Dave Francisco, Allyson Molzahn, Connie Cathleen Wilson, Ron Schunk, Trina Hughes, Bianca Sierra

**University of Florida, Gainesville, FL**, United States:

Mark A. Atkinson, Scott C. Brakenridge, Ricardo F. Ungaro, Brittany Roth Manning, Lyle Moldawer

**University of Florida, Jacksonville, FL**, United States:

Jordan Oberhaus, Faheem W. Guirgis

**University of South Florida, Tampa, FL**, United States:

Brittney Borresen, Matthew L. Anderson

**The University of Texas at Austin, Austin, TX**, United States:

Lauren I. R. Ehrlich, Esther Melamed, Cole Maguire, Dennis Wylie, Justin F. Rousseau, Kerin C. Hurley, Janelle N. Geltman, Nadia Siles, Jacob E. Rogers, Pablo Guaman Tipan.
