# Supplementary material for: Baseline predictors for 28-day COVID-19 severity and mortality among hospitalized patients: results from the IMPACC study
Source: Front Med (Lausanne). 2025 Jul 4;12:1604388. doi: 10.3389/fmed.2025.1604388 (PMC12271175; doi:10.3389/fmed.2025.1604388)
Supplement: Supplementary file 1 [file Data_Sheet_1.docx]

Supplementary Material

**Supplemental Table 1** Baseline demographic, clinical, and common laboratory characteristics.

|  | | Overall (n=1102) | Non-severe (TG1-3, n=797) | Severe  (TG4-5, n=305) | P value |
| --- | --- | --- | --- | --- | --- |
| Age at enrollment (years) | Median (IQR) | 59(49-69) | 57(46-67) | 64(54-71) | <0.001 |
| Sex, no (%) | Male (%) | 669(60.7) | 464 (58.2) | 205(67.2) | 0.006 |
| Race, no (%) | White people | 534 (48.5) | 375 (47.1) | 159(52.1) | <0.001 |
|  | Black people | 252 (22.9) | 208 (26.1) | 44 (14.4) |  |
|  | Asian people | 46 (4.2) | 34 (4.3) | 12 (3.9) |  |
|  | Other | 270 (24.5) | 180 (22.6) | 90 (29.5) |  |
| Ethnicity, no (%) | Hispanic or Latino | 346 (31.4) | 229 (28.7) | 117 (38.4) | <0.001 |
|  | Not Hispanic or Latino | 711 (64.5) | 544 (68.3) | 167 (54.8) |  |
|  | Unknown | 45 (4.1) | 25 (3.1) | 20 (6.7) |  |
| BMI category in Kg/m2, no. (%) | Underweight | 15 (1.4) | 14 (1.8) | 1 (0.3) | 0.017 |
|  | Normal weight | 155 (14.1) | 102 (12.8) | 53 (17.4) |  |
|  | Overweight  (25.1–29.9) | 281 (25.5) | 205 (25.7) | 76 (24.9) |  |
|  | Class 1-2 Obesity (30-39.9) | 452 (41.0) | 331 (41.5) | 121 (39.7) |  |
|  | Class 3 Obesity (40+) | 160 (14.5) | 110 (13.8) | 50 (16.4) |  |
|  | Unknown | 39 (3.5) | 35 (4.4) | 4 (1.3) |  |
| Symptom onset to hospitalization (days), no. (%) | 3 days or less | 226 (20.5) | 167 (21.0) | 59 (19.3) | 0.022 |
|  | 4-7 days | 308 (27.9) | 218 (27.4) | 90 (29.5) |  |
|  | 8-14 days | 288 (26.1) | 225 (28.2) | 63 (20.7) |  |
|  | More than 2 weeks | 80 (7.3) | 49 (6.1) | 31 (10.2) |  |
|  | Unknown | 200 (18.1) | 138 (17.5) | 62 (20.3) |  |
| Radiographic findings on chest imaging, no. (%) | No infiltrates | 280 (25.4) | 234 (29.4) | 46 (15.1) | <0.001 |
|  | Unilateral infiltrates | 96 (8.7) | 73 (9.2) | 23 (7.5) |  |
|  | Bilateral infiltrates | 660 (59.9) | 433 (54.3) | 227 (74.4) |  |
|  | Unknown | 66 (6.0) | 57 (7.2) | 9 (3.0) |  |
| SpO2/FiO2 at lowest saturation, mean(SD) | | 322.8(121.2) | 369.68 (87.18) | 200.37 (111.93) | <0.001 |
| SpO2 at lowest saturation, mean(SD) | | 90.56 (6.28) | 91.94 (4.03) | 86.95 (9.08) | <0.001 |
| FiO2 at lowest saturation, mean(SD) | | 0.36 (0.22) | 0.27 (0.12) | 0.57 (0.28) | <0.001 |
| Comorbidities, no. (%) | Hypertension | 645 (58.5) | 449 (56.3) | 196 (64.3) | 0.017 |
|  | Diabetes | 405 (36.8) | 270 (33.9) | 135 (44.3) | 0.001 |
|  | Pulmonary disease (excludes asthma) | 221 (20.1) | 148 (18.6) | 73 (23.9) | 0.047 |
|  | Asthma | 169 (15.3) | 127 (15.9) | 42 (13.8) | 0.372 |
|  | Chronic cardiac disease | 295 (26.8) | 206 (25.8) | 89 (29.2) | 0.263 |
|  | Chronic kidney disease (CKD) | 168 (15.2) | 117 (14.7) | 51 (16.7) | 0.399 |
|  | Malignant neoplasm | 113 (10.3) | 80 (10.0) | 33 (10.8) | 0.702 |
|  | Chronic neurological disorder | 130 (11.8) | 96 (12.0) | 34 (11.1) | 0.679 |
|  | Liver disease | 52 (4.7) | 35 (4.4) | 17 (5.6) | 0.408 |
|  | History of solid organ or bone marrow transplant | 66 (6.0) | 49 (6.1) | 17 (5.6) | 0.719 |
|  | HIV | 20 (1.8) | 18 (2.3) | 2 (0.7) | 0.075 |
|  | Current or former smoking and/or vaping | 364 (33.0) | 254 (31.9) | 110 (36.1) | 0.185 |
|  | Drug or alcohol abuse or cannabis use | 82 (7.4) | 60 (7.5) | 22 (7.2) | 0.858 |
| Symptoms, no. (%) | Any upper or lower respiratory | 960 (87.1) | 691 (86.7) | 269 (88.2) | 0.507 |
|  | Cough | 675 (61.3) | 501 (62.9) | 174 (57.0) | 0.076 |
|  | Cough with sputum production | 159 (14.4) | 124 (15.6) | 35 (11.5) | 0.084 |
|  | Cough with bloody sputum | 25 (2.3) | 21 (2.6) | 4 (1.3) | 0.187 |
|  | Sore throat | 94 (8.5) | 74 (9.3) | 20 (6.6) | 0.147 |
|  | Runny nose | 54 (4.9) | 40 (5.0) | 14 (4.6) | 0.768 |
|  | Wheezing | 59 (5.4) | 37 (4.6) | 22 (7.2) | 0.09 |
|  | Chest pain | 226 (20.5) | 178 (22.3) | 48 (15.7) | 0.015 |
|  | Shortness of breath | 794 (72.1) | 550 (69.0) | 244 (80.0) | <0.001 |
|  | Fever | 547 (49.6) | 413 (51.8) | 134 (43.9) | 0.019 |
|  | Chills/rigors/shivering | 276 (25.0) | 222 (27.9) | 54 (17.7) | <0.001 |
|  | Fatigue/malaise | 463 (42.0) | 357 (44.8) | 106 (34.8) | 0.003 |
|  | Myalgia | 262 (23.8) | 214 (26.9) | 48 (15.7) | <0.001 |
|  | Any neurologic | 343 (31.1) | 249 (31.2) | 94 (30.8) | 0.892 |
|  | Headache | 194 (17.6) | 153 (19.2) | 41 (13.4) | 0.025 |
|  | Confusion | 63 (5.7) | 31 (3.9) | 32 (10.5) | <0.001 |
|  | Syncope | 19 (1.7) | 14 (1.8) | 5 (1.6) | 0.894 |
|  | Anosmia | 113 (10.3) | 90 (11.3) | 23 (7.5) | 0.066 |
|  | Any gastrointestinal | 430 (39.0) | 340 (42.7) | 90 (29.5) | <0.001 |
|  | Abdominal pain | 110 (10.0) | 84 (10.5) | 26 (8.5) | 0.318 |
|  | Nausea/vomiting | 249 (22.6) | 207 (26.0) | 42 (13.8) | <0.001 |
|  | Diarrhea | 244 (22.1) | 195 (24.5) | 49 (16.1) | 0.003 |
|  | Joint pain | 43 (3.9) | 36 (4.5) | 7 (2.3) | 0.088 |
|  | Swelling | 29 (2.6) | 17 (2.1) | 12 (3.9) | 0.095 |
|  | Unable to walk | 25 (2.3) | 13 (1.6) | 12 (3.9) | 0.022 |
| Abnormal Common Laboratory tests, no. (%) | Lymphocyte count (<500/microliter) | 134 (12.2) | 68 (8.5) | 66 (21.6) | <0.001 |
|  | Platelets (<100,000/microliter) | 55 (5.0) | 31 (3.9) | 24 (7.9) | 0.007 |
|  | ALT (>1.5x site-specific upper limit of normal) | 189 (17.2) | 128 (16.1) | 61 (20.0) | 0.121 |
|  | Creatinine (>=1.5 mg/dL) | 178 (16.2) | 102 (12.8) | 76 (24.9) | <0.001 |
|  | CRP (>=10 mg/L) | 472 (42.8) | 327 (41.0) | 145 (47.5) | 0.051 |
|  | Abnormal D-dimer (>0.5 mg/L) | 564 (51.2) | 396 (49.7) | 168 (55.1) | 0.109 |
|  | Troponin (>0.4 ng/mL) | 91 (8.3) | 59 (7.4) | 32 (10.5) | 0.096 |

Two-sided two-sample t-tests are used to compare continuous variables between severe and non-severe groups, chi-squared tests are used for other comparisons. TG: trajectory group.

Supplemental Table 2 **Examining the performance of the SpO2/FiO2 model among sex, race, and ethnicity subgroups on all 1102 participants. Use SpO2/FiO2 = 285.5 (probability=0.5) as cut off.**

|  | Severe, n=305 | Sen., (%) | P value | | Non-severe, n=797 | Spec., (%) | P value | |
| --- | --- | --- | --- | --- | --- | --- | --- | --- |
| Sex | | | | | | | | |
| Female | 100 | 75 | 0.23 | | 333 | 83.5 | 0.375 | |
| Male | 205 | 81 |  |  | 464 | 81 |  |  |
| Race | | | | | | | | |
| Black people | 44 | 72.7 | 0.31 | 0.454 | 208 | 87 | 0.0568 | 0.074 |
| White people | 159 | 79.9 | Ref |  | 375 | 80.8 | Ref |  |
| Asian people | 12 | 66.7 | 0.287 |  | 34 | 70.6 | 0.1588 |  |
| Other | 90 | 82.2 | 0.652 |  | 180 | 81.1 | 0.9304 |  |
| Ethnicity | | | | | | | | |
| Hispanic or Latino | 117 | 83.8 | 0.0739 | 0.1365 | 229 | 79.5 | 0.1469 | 0.0656 |
| Not Hispanic or Latino | 167 | 74.9 | Ref |  | 544 | 83.8 | Ref |  |
| Unknown | 21 | 85.7 | 0.2797 |  | 24 | 66.7 | 0.0337 |  |

Ref: reference class. Sens.: sensitivity. Spec.: specificity. Logistic regression is used to test the significance between the reference class and other classes. The likelihood ratio test is used to test the significance of the entire subgroup, p<0.05 is considered significant.

**Supplemental Table 3** Lasso selected features for noninvasive clinical features + top 4 laboratory features in predicting 28-day **severity**.

| Dataset  (candidate variables, n) | Predictors (imputed missing% in the training set) | Train AUC | Severe,n (Total,n) | Test AUC | Severe,n (Total,n) |
| --- | --- | --- | --- | --- | --- |
| Noninvasive clinical (see supplemental Table 1) | SpO2/FiO2 | 0.865 | 186(658) | 0.874 | 119(444) |
|  | +age | 0.88 |  | 0.875 |  |
|  | +SpO2 | 0.883 |  | 0.882 |  |
|  | +confusion | 0.887 |  | 0.881 |  |
|  | +CKD | 0.89 |  | 0.878 |  |
| Clinical with seven common laboratory tests (Supplemental Table 1) | Age+S/F+BMI | 0.885 | 186(658) | 0.884 | 114(426) |
|  | + Creatinine(3.34) | 0.894 |  | 0.885 |  |
|  | +platelets(4.56) | 0.898 |  | 0.887 |  |
|  | + Asian | 0.9 |  | 0.878 |  |
|  | +lymph(<500/microliter) | 0.902 |  | 0.877 |  |
| Clinical+ Olink(92) | Age+S/F+BMI | 0.884 | 177(627) | 0.885 | 115(426) |
|  | + FGF23 | 0.899 |  | 0.896 |  |
|  | +IL6 | 0.909 |  | 0.9 |  |
|  | + LTA | 0.922 |  | 0.916 |  |
|  | + IL17C | 0.926 |  | 0.912 |  |
| Clinical + CyTOF  (66) | Age+S/F+BMI | 0.892 | 122(439) | 0.89 | 69(291) |
|  | + CD4+ T Cell (CM) | 0.906 |  | 0.883 |  |
|  | + B Cell (Plasmablast) | 0.909 |  | 0.878 |  |
|  | + respiratory symptom | 0.916 |  | 0.871 |  |
|  | + platelets (4.78) | 0.92 |  | 0.871 |  |
| Clinical + Global Plasma Metabolomics (658) | Age+S/F+BMI | 0.886 | 177(599) | 0.875 | 109(399) |
|  | + S-allylcysteine | 0.909 |  | 0.878 |  |
|  | + uridine | 0.922 |  | 0.88 |  |
|  | + omeprazole | 0.927 |  | 0.878 |  |
|  | + X100009002 | 0.93 |  | 0.878 |  |
| Clinical +Proteomics-targeted (203) | Age+S/F+BMI | 0.893 | 174(585) | 0.873 | 102(384) |
|  | + platelets(4.96) | 0.899 |  | 0.876 |  |
|  | +lymph(<500/microliter) | 0.902 |  | 0.872 |  |
|  | + Creatinine(3.76) | 0.902 |  | 0.875 |  |
|  | + symptom on set to hospital(>2 weeks) | 0.905 |  | 0.88 |  |
| Clinical + Nasal metagenomics counts (1679) | Age+S/F+BMI | 0.885 | 154(558) | 0.889 | 100(359) |
|  | + Creatinine(3.05) | 0.895 |  | 0.891 |  |
|  | + platelets(3.94) | 0.898 |  | 0.896 |  |
|  | + any drug/alcohol/ cannabis use | 0.9 |  | 0.89 |  |
|  | + Dyadobacter | 0.904 |  | 0.887 |  |
| Clinical + Nasal viral load (20) | Age+S/F+BMI | 0.885 | 134(474) | 0.893 | 89(309) |
|  | + Creatinine(3.59) | 0.897 |  | 0.895 |  |
|  | + CKD | 0.902 |  | 0.893 |  |
|  | +asthma | 0.905 |  | 0.881 |  |
|  | + platelets (4.22) | 0.908 |  | 0.888 |  |
| Clinical + Serum autoantibody (3) | Age+S/F+BMI | 0.887 | 160(567) | 0.895 | 95(357) |
|  | + platelets (4.76) | 0.893 |  | 0.899 |  |
|  | +lymph(<500/microliter) | 0.897 |  | 0.894 |  |
|  | + symptom on set to hospital(>2 weeks) | 0.899 |  | 0.898 |  |
|  | + Asian | 0.904 |  | 0.89 |  |
| Clinical + Serum RBD abtiters(4) | Age+S/F+BMI | 0.879 | 171(613) | 0.884 | 108(400) |
|  | + Creatinine (2.94) | 0.887 |  | 0.886 |  |
|  | + platelets (3.26) | 0.893 |  | 0.889 |  |
|  | +lymph(<500/microliter) | 0.894 |  | 0.887 |  |
|  | + Not Hispanic or Latino | 0.898 |  | 0.889 |  |
| Clinical + Serum sarscov2 abtiters (3745) | Age+S/F+BMI | 0.887 | 174(614) | 0.884 | 110(398) |
|  | + Creatinine (3.26) | 0.896 |  | 0.885 |  |
|  | +frag2 | 0.9 |  | 0.883 |  |
|  | +frag218 | 0.902 |  | 0.88 |  |
|  | +frag4 | 0.907 |  | 0.88 |  |
| Clinical + Nasal transcriptomics (58302) | Age+S/F+BMI | 0.878 | 148(514) | 0.887 | 99(347) |
|  | +ENSG00000261816 | 0.896 |  | 0.884 |  |
|  | + Creatinine (3.11) | 0.904 |  | 0.885 |  |
|  | +ENSG00000252633 | 0.914 |  | 0.874 |  |
|  | + ENSG00000273218 | 0.924 |  | 0.853 |  |
| Clinical + PBMC transcriptomics (58302) | Age+S/F+BMI | 0.885 | 165(578) | 0.871 | 101(380) |
|  | +ENSG00000054392 | 0.902 |  | 0.878 |  |
|  | +ENSG00000280080 | 0.907 |  | 0.871 |  |
|  | +ENSG00000105808 | 0.91 |  | 0.865 |  |
|  | +ENSG00000271119 | 0.912 |  | 0.861 |  |

S/F: SpO2/FiO2 at lowest saturation

Frag2: HCoV229E__N__NP_073556.1__frag__2

Frag218: InfectiousBronchitisCoV__1ab__NP_066134.1__frag__218

Frag4: HCoV229E__HCoV229Egp1__NP_073549.1__frag__4

LTA: Lymphotoxin-alpha.

CKD: Chronic kidney disease.

X100009002: 1-(1-enyl-palmitoyl)-2-arachidonoyl-GPE (P-16:0/20:4)

Selected variables containing missing values are continuous Creatinine and Platelets. The percentages of missing values of the three variables are all less than 5% within the training set. These missing values were imputed using the missForest algorithm before feature selection. Testing datasets are not imputed, observations with missing selected features are removed from the testing dataset. For each row, a multivariable logistic regression is built on the balanced training data using the features in the row and all features above the row within each merged dataset.

**Supplemental Table 4** Lasso selected features for clinical features + laboratory features in predicting 28-day mortality.

| Dataset  (candidate variables, n) | Predictors | Train AUC | Death,n (Total,n) | Test AUC | Death,n (Total,n) |
| --- | --- | --- | --- | --- | --- |
| Noninvasive clinical | SpO2/FiO2 | 0.735 | 57(658) | 0.78 | 42(444) |
|  | +age | 0.802 |  | 0.82 |  |
|  | +asthma | 0.82 |  | 0.72 |  |
|  | +CKD | 0.837 |  | 0.73 |  |
|  | +FiO2 | 0.839 |  | 0.724 |  |
| Clinical including seven common laboratory tests | Age+S/F+BMI | 0.827 | 57(658) | 0.833 | 41(430) |
|  | + Creatinine (>=1.5 mg/dL) | 0.848 |  | 0.843 |  |
|  | +platelets | 0.864 |  | 0.855 |  |
|  | + asthma | 0.874 |  | 0.782 |  |
|  | +CKD | 0.879 |  | 0.78 |  |
| Clinical+ Olink(92) | Age+S/F+BMI | 0.829 | 54(627) | 0.828 | 41(426) |
|  | + TNFRSF11B | 0.883 |  | 0.863 |  |
|  | +IL6 | 0.894 |  | 0.848 |  |
|  | + FGF23 | 0.895 |  | 0.85 |  |
|  | +IL10 | 0.9 |  | 0.849 |  |
| Clinical + CyTOF  (66) | Age+S/F+BMI | 0.844 | 36(439) | 0.83 | 24(301) |
|  | + Monocytes (CD14+CD16-) | 0.864 |  | 0.794 |  |
|  | + CD4+ Treg (CD39low) | 0.895 |  | 0.748 |  |
|  | + Chronic neurological disorder | 0.906 |  | 0.768 |  |
|  | + asthma | 0.914 |  | 0.75 |  |
| Clinical + Global Plasma Metabolomics (658) | Age+S/F+BMI | 0.824 | 52(599) | 0.828 | 38(399) |
|  | + ribitol | 0.889 |  | 0.874 |  |
|  | + quinolinate | 0.9 |  | 0.885 |  |
|  | + phenyllactate (PLA) | 0.904 |  | 0.887 |  |
|  | + N1-Methyl-2-pyridone-5-carboxamide | 0.908 |  | 0.885 |  |
| Clinical +Proteomics-targeted (203) | Age+S/F+BMI | 0.826 | 52(585) | 0.836 | 38(384) |
|  | + platelets | 0.862 |  | 0.843 |  |
|  | +CKD | 0.873 |  | 0.85 |  |
|  | +asthma | 0.883 |  | 0.779 |  |
|  | + Creatinine | 0.885 |  | 0.783 |  |
| Clinical + Nasal metagenomics counts (1679) | Age+S/F+BMI | 0.832 | 49(558) | 0.834 | 38(362) |
|  | +CKD | 0.855 |  | 0.826 |  |
|  | +asthma | 0.866 |  | 0.748 |  |
|  | + Creatinine(>=1.5 mg/dL) | 0.869 |  | 0.76 |  |
|  | + platelets | 0.878 |  | 0.782 |  |
| Clinical + Nasal viral load (20) | Age+S/F+BMI | 0.823 | 46(474) | 0.836 | 33(311) |
|  | +CKD | 0.854 |  | 0.836 |  |
|  | + Creatinine (>=1.5 mg/dL) | 0.859 |  | 0.835 |  |
|  | +asthma | 0.865 |  | 0.789 |  |
|  | + platelets | 0.874 |  | 0.801 |  |
| Clinical + Serum autoantibody (3) | Age+S/F+BMI | 0.832 | 49(567) | 0.821 | 35(354) |
|  | + platelets | 0.864 |  | 0.838 |  |
|  | +asthma | 0.883 |  | 0.728 |  |
|  | +CKD | 0.891 |  | 0.737 |  |
|  | + Creatinine | 0.893 |  | 0.738 |  |
| Clinical + Serum RBD abtiters(4) | Age+S/F+BMI | 0.829 | 55(613) | 0.83 | 40(404) |
|  | + platelets | 0.858 |  | 0.835 |  |
|  | +CKD | 0.871 |  | 0.839 |  |
|  | + Creatinine (>=1.5 mg/dL) | 0.871 |  | 0.846 |  |
|  | +asthma | 0.879 |  | 0.78 |  |
| Clinical + Serum sarscov2 abtiters (3745) | Age+S/F+BMI | 0.827 | 53(614) | 0.825 | 41(409) |
|  | +frag290 | 0.86 |  | 0.824 |  |
|  | +frag325 | 0.863 |  | 0.818 |  |
|  | +frag52 | 0.869 |  | 0.817 |  |
|  | +frag11 | 0.884 |  | 0.817 |  |
| Clinical + Nasal transcriptomics (58302) | Age+S/F+BMI | 0.824 | 48(514) | 0.836 | 39(357) |
|  | +ENSG00000261816 | 0.863 |  | 0.836 |  |
|  | +ENSG00000270882 | 0.879 |  | 0.82 |  |
|  | +ENSG00000244006 | 0.888 |  | 0.818 |  |
|  | +ENSG00000211746 | 0.9 |  | 0.813 |  |
| Clinical + PBMC transcriptomics (58302) | Age+S/F+BMI | 0.819 | 51(578) | 0.833 | 35(371) |
|  | +ENSG00000259803 | 0.859 |  | 0.827 |  |
|  | +asthma | 0.885 |  | 0.663 |  |
|  | +platelets | 0.904 |  | 0.66 |  |
|  | +ENSG00000007968 | 0.909 |  | 0.667 |  |
| Clinical + Global Plasma Metabolomics +Olink | Age+S/F+BMI(ASB) | 0.827 | 49(578) | 0.826 | 38(391) |
|  | ASB+ ribitol | 0.887 |  | 0.873 |  |
|  | ASB+ TNFRSF11B | 0.88 |  | 0.86 |  |
|  | ASB+ ribitol+ TNFRSF11B | 0.9 |  | 0.88 |  |

Frag290: HCoV229E__HCoV229Egp1__NP_073549.1__frag__290

Frag325: SARS1__orf1ab__NP_828849.2__frag_325

Frag52: HCoVOC43__orf1ab__YP_009555238.1__frag__52

Frag11 : SARS1__sars3a__NP_828852.2__frag_11

TNFRSF11B: tumor necrosis factor receptor superfamily member 11B

FGF23: Fibroblast growth factor 23

CKD: Chronic kidney disease.

S/F: SpO2/FiO2 at lowest saturation

For each row, a multivariable logistic regression is built on the balanced training data using the features in the row and all features above the row within each merged dataset.

**Supplemental Table** 5: Multivariable logistic regressions for 28-day in-hospital mortality derived from the balanced training set. Reference level of BMI is “normal”. Clinical model is SpO2/FiO2 +age+ BMI.

|  | Clinical Model + TNFRSF11B | Clinical Model + ribitol |
| --- | --- | --- |
| Train AUC (95%CI, n) | 0.883 (0.841-0.926, n=627) | 0.889 (0.856-0.922, n=599) |
| Test AUC (95%CI, n) | 0.863 (0.812-0.914, n=426) | 0.874 (0.822-0.927, n=399) |
| Intercept | -17.64 | -2.7 |
| SpO2/FiO2 | -0.0064 | -0.00701 |
| Age | 0.0514 | 0.07934 |
| Underweight | -18.23 | -19.6 |
| Overweight | -1.34 | -1.66 |
| Class 1-2 Obesity (30-39.9) | -0.76 | -1.17 |
| Class 3 Obesity (40+) | 0.13 | -0.65 |
| Unknown BMI | -17.1 | -19.65 |
| TNFRSF11B | 1.529 | - |
| ribitol | - | 3.589 |

**Supplemental Table** 6: Comparison of ribitol between groups for males and females.

| Sex | Group (n) | Normalized Ribitol, mean(sd) | P value |
| --- | --- | --- | --- |
| Male | Dead (62) | 0.277(0.37) | <0.001 |
|  | Alive (527) | -0.045(0.32) |  |
|  | Nonsevere (411) | -0.086 (0.3) | <0.001 |
|  | Severe (188) | 0.151 (0.36) |  |
| Female | Dead (28) | 0.503 (0.64) | <0.001 |
|  | Alive (371) | -0.065 (0.39) |  |
|  | Nonsevere (301) | -0.102 (0.37) | <0.001 |
|  | Severe (98) | 0.211 (0.52) |  |
| Male | Severe | 0.151 (0.36) | 0.3 |
| Female |  | 0.211 (0.52) |  |
| Male | Nonsevere | -0.086 (0.3) | 0.53 |
| Female |  | -0.102 (0.37) |  |
| Male | Dead | 0.277(0.37) | 0.09 |
| Female |  | 0.503 (0.64) |  |
| Male | Alive | -0.045(0.32) | 0.41 |
| Female |  | -0.065 (0.39) |  |

Two sample t-test was used to compare variables between groups.


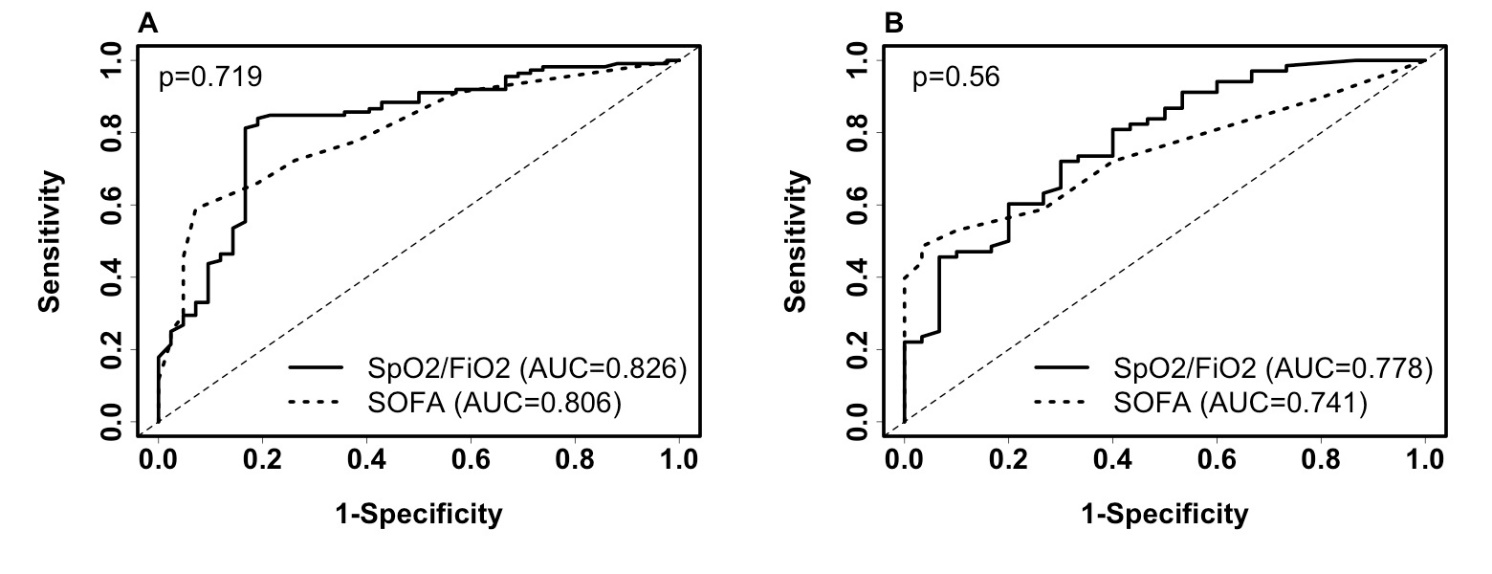


**Supplemental Figure 1** Comparison of ROC curves of SpO2/FiO2 and SOFA for predicting 28-day COVID-19 severity among ICU patients. **A.** ROC on the training set (severe, n=112; non-severe, n=42). SpO2/FiO2: AUC=0.826 (95% CI: 0.7485-0.9029, sensitivity=87.5%, specificity=57.1%, probability cut-off=0.5, i.e. SpO2/FiO2=285.5). SOFA score: AUC=0.806(95% CI: 0.7344-0.8781, sensitivity=72.3%, specificity=73.8%). **B.** ROC on the Testing set (severe, n=68; non-severe, n=30). SpO2/FiO2: AUC=0.778(95% CI: 0.679-0.8774, sensitivity=89.7%, specificity=46.7%). SOFA score: AUC=0.741(95% CI: 0.6471-0.8358, sensitivity=72.1%, specificity=60%). Paired Delong’s test was used to obtain p values.


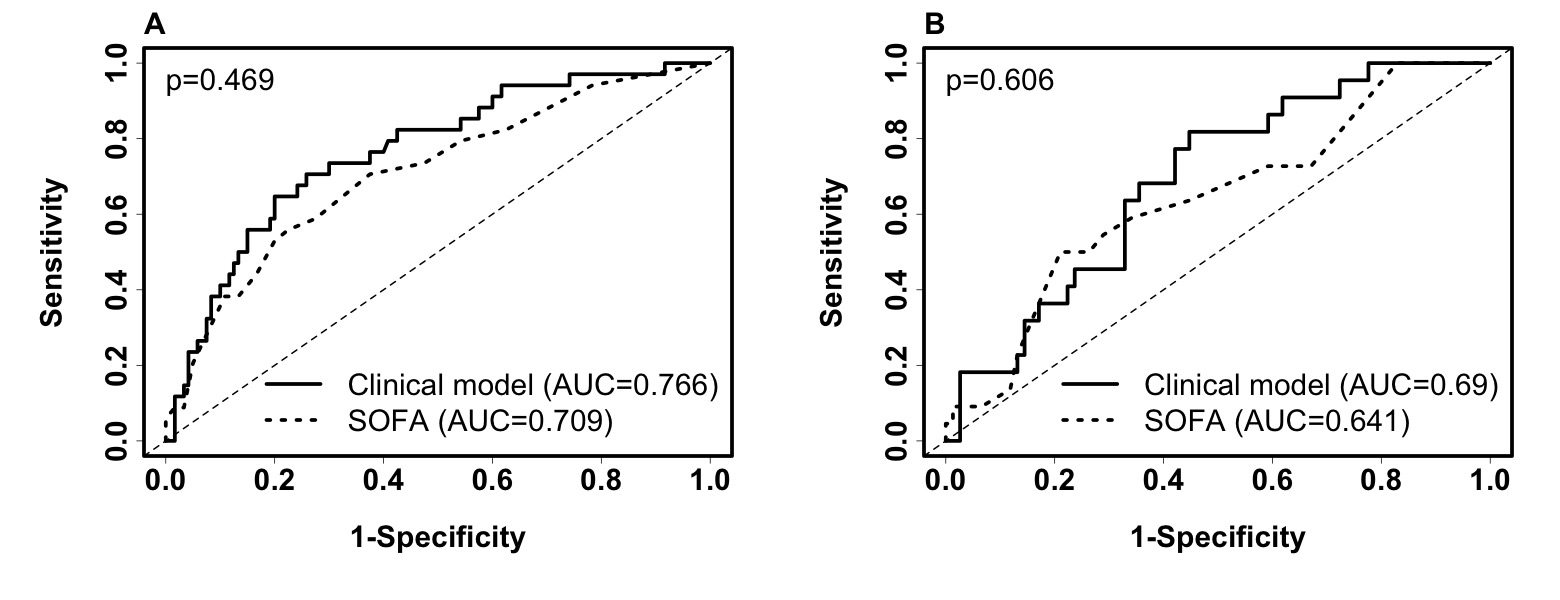


**Supplemental Figure** **2** Comparison of ROC curves of clinical model (SpO2/FiO2+age+BMI) and SOFA for predicting 28-day in-hospital mortality among ICU patients. **(A).** ROC on the training set (Death, n=34; Alive, n=120). Clinical model: AUC=0.766(95% CI: 0.6753-0.8563, sensitivity=85.3%, specificity=43.3%), SOFA score: AUC=0.709(95% CI: 0.6077-0.8107, sensitivity=73.5%, specificity=52.5%). **(B).** ROC on the Testing set (Death, n=22; Alive, n=76). Clinical model: AUC=0.69(95% CI: 0.5737-0.8055, sensitivity=81.8%, specificity=52.6%), SOFA score: AUC=0.641(95% CI: 0.5085-0.7726, sensitivity=63.6%, specificity=55.3%). (probability cut off = 0.5). Paired Delong’s test was used to obtain p values.
